# Accurate Estimation of Yield Strength and Ultimate Tensile Strength through Instrumented Indentation Testing and Chemical Composition Testing

**DOI:** 10.3390/ma15030832

**Published:** 2022-01-22

**Authors:** Martin Scales, Joel Anderson, Jeffrey A. Kornuta, Nathan Switzner, Ramon Gonzalez, Peter Veloo

**Affiliations:** 1 Exponent, Inc., Houston, TX 77042, USA; mscales@exponent.com; 2 RSI Pipeline Solutions, New Albany, OH 43054, USA; janderson@rsi-ps.com (J.A.); nswitzner@rsi-ps.com (N.S.); 3 Pacific Gas & Electric Company, Walnut Creek, CA 94598, USA; r4gg@pge.com (R.G.); pxvz@pge.com (P.V.)

**Keywords:** IIT, nondestructive testing, integrity, pipelines, materials verification

## Abstract

Federal rule changes governing natural gas pipelines have made non-destructive techniques, such as instrumented indentation testing (IIT), an attractive alternative to destructive tests for verifying properties of steel pipeline segments that lack traceable records. Ongoing work from Pacific Gas and Electric Company’s (PG&E) materials verification program indicates that IIT measurements may be enhanced by incorporating chemical composition data. This paper presents data from PG&E’s large-scale IIT program that demonstrates the predictive capabilities of IIT and chemical composition data, with particular emphasis given to differences between ultimate tensile strength (UTS) and yield strength (YS). For this study, over 80 segments of line pipe were evaluated through tensile testing, IIT, and compositional testing by optical emission spectroscopy (OES) and laboratory combustion. IIT measurements of UTS were, generally, in better agreement with destructive tensile data than YS and exhibited about half as much variability as YS measurements on the same sample. The root-mean squared error for IIT measurements of UTS and YS, respectively, were 27 MPa (3.9 ksi) and 43 MPa (6.2 ksi). Next, a machine learning model was trained to estimate YS and UTS by combining IIT with chemical composition data. The agreement between the model’s estimated UTS and tensile UTS values was only slightly better than the IIT-only measurements, with an RMSE of 21 MPa (3.1 ksi). However, the YS estimates showed much greater improvement with an improved RMSE of 27 MPa (3.9 ksi). The experimental, mechanical, and metallurgical factors that contributed to IIT’s ability to consistently determine destructive UTS, and the differences in its interaction with composition as compared to YS, are discussed herein.

## 1. Introduction

In traditional structural design, in-service loads are estimated and compared with known material properties to determine an appropriate structural configuration. However, integrity verification of existing structures sometimes presents the inverse problem where configuration is known but the material properties are unknown. Estimating the mechanical properties of in-service materials is essential in such cases and underpins recent changes in regulations governing pipelines in the United States [1].

These regulatory changes for natural gas transmission pipelines have prompted operators to deploy nondestructive testing (NDT) technologies to verify material properties when traceable, verifiable, and complete (TVC) records do not exist. Pertinent material properties for pipelines include yield strength (YS), ultimate tensile strength (UTS), fracture toughness, hardness, chemical composition, and metallographically-determined grain size and phase fractions. All these parameters can be used to estimate the steel grade (per [2]) for subsequent calculations and for pipeline integrity management.

Instrumented indentation testing (IIT) has, thus, become a prominent technique for NDT in the pipeline community, although the specific implementation of the technique varies a great deal. A method involving a single indentation of pipeline steels and microscopy of the residual deformation was presented in [3] and updated in [4]. Those authors also explored various indentation load levels in assessing degradation of API pipeline samples in [5]. A combination of indentations, with a flat indenter and numerical analysis, was used in [6] in extracting the full hardening curve from grade X80 pipe, with validation against tensile data. The same authors used the technique to assess the mechanical properties of welds in X80 pipe in [7], and an overall review of the flat indenter technique was presented in [8]. IIT has also been used to measure residual stresses in steels [9].

The Pacific Gas and Electric Company (PG&E, Walnut Creek, CA, USA) has adopted IIT for nondestructively estimating YS and UTS. PG&E has spent several years developing an IIT program for its engineering critical assessment activities (see [10,11,12,13,14]). This effort has included destructive tensile testing of some pipe features for direct comparison to the IIT measurements (a pipe feature is any contiguous section of pipe between girth welds). Specifics of the adopted technique are discussed in Section 2.1.

Since the program launched in late 2016, over 5000 IIT measurements have been obtained across nearly 200 unique line pipe features, spanning a wide range of vintages, making the dataset presented in this work the largest collection of IIT data on line pipe steels known to the authors. IIT’s estimate of UTS is particularly robust, exhibiting reduced scatter compared to previously published correlations of hardness data with UTS [15]. Additionally, whereas hardness has typically been used to estimate only UTS, IIT can be used to nondestructively measure both UTS and YS.

The strength of steel is known to be strongly influenced by chemical composition. The base strength of nearly-pure ferritic iron can be relatively low, about 85 MPa [16]. Therefore, steelmaking incorporates one or more approaches to increase strength, including alloying, controlled cooling, grain size reduction, strain hardening, and precipitation hardening. The strengthening methods, other than alloying, are not assessed in this article because they are not easily quantified in the field. For chemical composition estimation (i.e., elemental mass fraction) of line-pipe steels, however, PG&E established reliable methods and relationships between destructive laboratory chemical composition results and non-destructive composition estimates [17,18].

Line pipe steels of low and moderate strength are typically low-carbon, ferrite-pearlite steels. Manganese is added to reduce impurities, and both carbon and manganese increase hardness [19]. In the 1930s, Bain estimated that manganese increases the UTS of ferrite-pearlite steels by about 25 MPa for each 1 weight percent (wt.%) Mn addition [20]. Nearly 50 years later, Pickering, and coauthors from British Steel, reported in [21,22] that manganese increases YS and published the following empirical relationship:

(1) $$\mathrm{YS}\left(\mathrm{MPa}\right)=88+37\mathrm{Mn}+83\mathrm{Si}+2918N_{f}+15.1d^{-\frac{1}{2}}$$

where manganese (Mn) and silicon (Si) are specified in wt.%, $N_{f}$ is free nitrogen, and d is average grain size (in mm). Equation (1) applies only for low-carbon ferrite-pearlite steels, and the reported accuracy of Equation (1) was ±31 MPa (4.5 ksi). Both manganese and carbon increase UTS, as observed by the following relationship developed by the same authors, because carbon makes up 0.77 wt.% of the microstructural constituent, *pearlite*, where *pearlite* represents the percent pearlite observed via metallographic evaluation [19]:

(2) $$\mathrm{UTS}\left(\mathrm{MPa}\right)=294+28\mathrm{Mn}+83\mathrm{Si}+3.85\left(pearlite\right)+7.7d^{-\frac{1}{2}}$$

Recent findings [23] have shown that, over the past 100 years, manganese content and the content of microalloying elements such as vanadium, titanium, niobium, chromium, and molybdenum in line pipe steels has tended to increase, while carbon content has tended to decrease. Therefore, these long-accepted empirical relationships in Equations (1) and (2) may not hold for some contemporary line pipe steels. Researchers have recently attempted to use machine learning to establish improved estimates of mechanical properties from chemical composition by coupling it with indentation data. In [24], a neural network model was created to determine hardness using a combination of processing parameters and chemical composition. It was found that the composition data were far more influential in accurately estimating product hardness than the processing parameters were. A genetic algorithm was used in [25] to determine the mechanical properties from Vickers indentation load-depth responses in steels. A comprehensive examination of input parameters and multiple machine learning techniques was presented in [26] with the objective of predicting ultimate strength and elongation, although the focus was on tailoring composition and processing to produce an intended result.

The work presented in this paper stands apart in that chemical composition data is used to further enhance the accuracy of mechanical properties, derived from IIT measurements, using multivariate linear regressions that were trained on benchmark destructive tensile data. Taken together, IIT and chemical composition analyses give reliable nondestructive estimates of strength, particularly UTS. This paper presents data collected by PG&E that shows IIT measurements of UTS exhibit about half as much variability as YS. Chemical composition data and its role in bolstering the raw IIT measurements through incorporation into a linear regression model, trained against benchmark tensile data, are presented as well. IIT alone, and IIT together with the chemical composition effect, are also directly compared to destructive tensile measurements. This paper provides a method and model by which users of low carbon steels can use non-destructive IIT and chemical composition information to determine YS and UTS with improved accuracy.

## 2. Materials and Methods

### 2.1. Instrumented Indentation Testing

While many implementations of IIT exist, they all involve an indentation process during which a shaped indenter is brought into contact with the material of interest through one or more load and unload cycles. The normal force and displacement are recorded throughout the indentation process, and the tested materials’ mechanical properties are estimated from the load-depth response. As described in the international standard governing IIT [27], IIT sets itself apart from traditional hardness testing through this continuous recording of load and depth. Doing so enables extraction of a multitude of mechanical properties and obviates the need for subsequent optical inspection of the indent.

**Figure 1:**
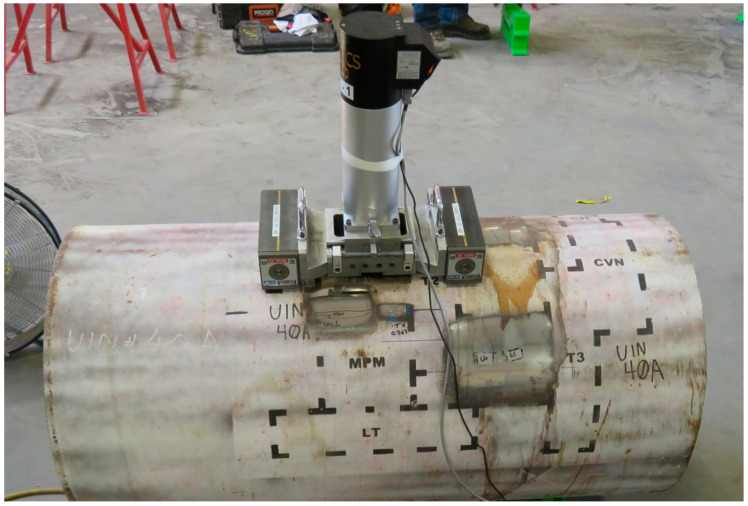
Photograph of the Frontics AIS 2100 IIT instrument mounted to a pipe sample.

The Frontics AIS 2100 IIT instrument, which was originally developed by engineers at Seoul National University (SNU, Seoul, Korea) (see [28,29,30,31]), was used in this work, and their loading scheme, as described below, was adopted. This tool employed a 0.5-mm diameter spherical indenter. A photograph of the Frontics AIS 2100 tool, mounted to a pipe sample, is shown in Figure 1. The instrument is shown mounted to a 24-inch diameter (61-cm; note that the pipelines tested in this effort were manufactured in the United States according to imperial units of measure) pipe by means of a magnetic mounting stand; the instrument was secured to the stand on flat ground, and then, the whole fixture was secured to the pipe by engaging the magnets. For pipes with outer diameters (OD) between 8 and 24 inches (20 and 61 cm), the instrument is mounted via a heavy-duty roller chain that is tightened to a specific torque. Secure mounting of the instrument is critical to ensure a sufficiently stiff test setup.

**Figure 2:**
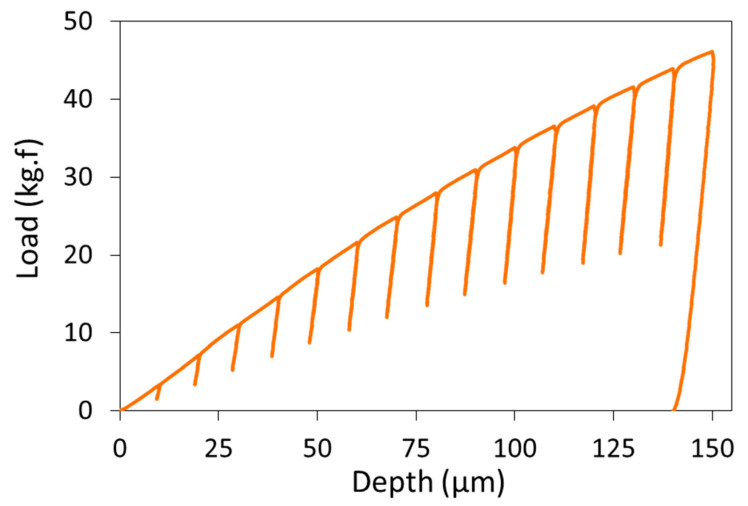
Example of a full load-depth response from an IIT measurement.

The tool was operated in displacement control and indented the sample surface to a maximum depth of 150 μm, through a series of 15 sequential load/partial-unload cycles, in increments of 10 μm. In each cycle, the indenter reached its target depth (e.g., 50 μm during the 5th load cycle) and unloaded to 50% of the force, corresponding to the target depth, before commencing the next load cycle and re-loading to the next target depth (e.g., 60 μm). As described below, these 15 discrete points of the load-depth response are used to obtain the stress and strain at these times, as well as the complete work hardening response. The resulting load-depth curve, at the conclusion of 15 cycles from a representative IIT measurement, is depicted in Figure 2.

The algorithm for extracting stress and strain, and subsequently YS and UTS, from this load-depth response is detailed in [28] through [31], and it is presented here in summary form. However, it is noted that those authors also relied on the pioneering work of Tabor [32].

**Figure 3:**
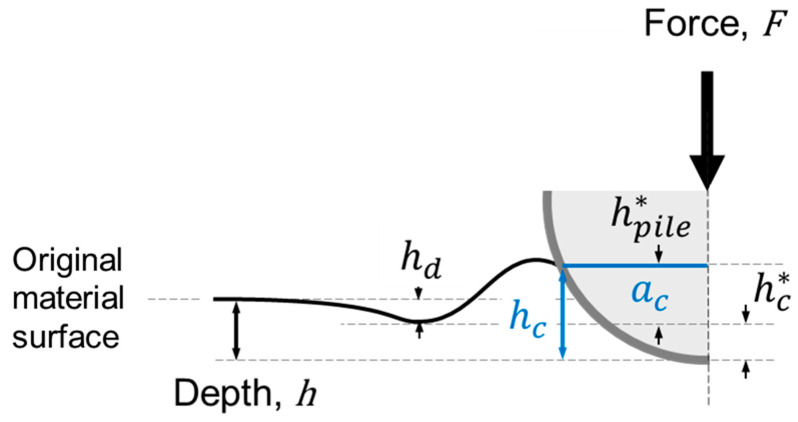
Schematic representation of a spherical indentation test and basic nomenclature; contact radius, $a_{c}$, and contact depth, $h_{c}$, highlighted in blue for clarity.

The representative stress of indentation, $\sigma_{r}$, is a function of the applied load, F, and the projected contact area of the indentation, $A_{c}$, as shown in Equation (3). $A_{c}$ can be calculated from the chordal radius $a_{c}$ of the indentation (see Equation (4) and Figure 3). Ψ is assigned a value of 3.0 based on the work of Jeon et al. [30].

(3) $$\sigma_{r}=\frac{1}{\Psi }\left(\frac{F}{A_{c}}\right)$$

(4) $$A_{c}=\pi a_{c}^{2}$$

The representative strain, $\epsilon_{r}$, is calculated according to Equation (5), in which the coefficient, α, is assigned a value of 0.14 based on experiments and analysis. It is worth mentioning that this equation, published in [28,30], differs from the relationship first proposed by Tabor in [32].

(5) $$\epsilon_{r}=\alpha \left(\frac{a_{c}}{R-h_{c}}\right)=\alpha \tan\gamma$$

Equations (1) through (3) introduce new unknowns: the contact radius $a_{c}$, and the contact depth $h_{c}$. These parameters, themselves, are difficult to measure directly in-situ and are, therefore, estimated afterwards from other measured quantities through an empirical model. The detailed procedure for extracting mechanical properties from the indentation response is presented in [28,31]. In brief, a power-law stress-strain relationship is assumed (Equation (6)), and the strain hardening exponent n is empirically related to the pile up around the indenter through Equation (7).

(6) $$\sigma =K\epsilon^{n}$$

(7) $$\frac{h_{pile}^{*}}{h-\omega \frac{F}{S_{i}}}=a\left(1+b_{1}n+b_{2}n^{2}\right)\left[1+c_{1}\frac{h}{R}+c_{2}{\left(\frac{h}{R}\right)}^{2}\right]$$

The numerical values of the parameters appearing in Equation (7) were established in [29] through numerical calibration of the IIT algorithm on a range of metallic materials. These parameters are presented here in Table 1.

An initial trial value for the hardening exponent *n* is assumed and used to update the contact parameters $a_{c}$ and $h_{c}$ through Equation (7). This new ratio subsequently changes the estimated representative stress and strain ($\sigma_{r}$ and $\epsilon_{r}$) at the 15 load maxima points from the IIT measurement. A power-law is fit to the final 10 of these updated points, and the process is repeated until the exponent *n* converges. Note that only the final 10 of the 15 stress-strain points are used in this study since, from the authors’ experience, the first 5 exhibit greater variability due to the very small loads achieved in those load cycles.

The instrument records the time of each instance of load and depth, and therefore, the strain rate can be established from the strain at the 10 discrete points. While the rate varies depending on strains achieved in each test, the typical strain rate over the final 10 of the 15 load maxima was between $2.5\times 10^{-3}\;s^{-1}$ and $0.5\times 10^{-3}\;s^{-1}$. This rate, however, is not fully analogous to the strain rate under uniaxial tensile loading. First, in general, the state of strain beneath the indenter in an IIT test is compressive and multiaxial. Second, the strains achieved just beneath the indenter during IIT exceed the strains reached in tensile tests due to the early onset of necking in tensile tests.

YS and UTS are then computed from the power-law stress-strain curve. In this work, YS is defined as

(8) $$\mathrm{YS}=K{\left(0.005\right)}^{n}$$

where YS, corresponding to a total strain of 0.5%, is consistent with the definition adopted for line pipe steels in [2]. This definition of YS has an advantage over alternatives, such as the 0.2% offset yield stress, in that it can be computed without first measuring Young’s Modulus. By the same token, this definition is only acceptable for materials with Young’s Modulus within a certain range—for example, some aluminum alloys may not have begun to yield at 0.5% strain. However, for the line pipe steels under consideration in this work, 0.5% strain is always beyond the elastic limit.

The UTS is taken as the stress at a strain numerically equal to the computed power-law hardening exponent, i.e., $\sigma (\epsilon_{\mathrm{UTS}}=n)$. This equality is derived from the Considère analysis of an incompressible, power-law material under uniaxial loading. Therefore, this approach assumes the deformation beneath the indenter is approximated by a uniaxial strain state. Thus, with the power-law stress-strain relationship established:

(9) $$\mathrm{UTS}=K{\left(n\right)}^{n}$$

Typically, at least eight measurements, in each of two different locations on the same pipe sample, are taken. This is partly motivated by regulatory requirements [1], but it is also done to permit detailed analysis of the uncertainty in the technique, which stems from the physical measurements and the iterative IIT algorithm that was adopted. Random error was further mitigated by this replicate sampling.

Through years of experience with this methodology, PG&E has developed several criteria that are used to filter out erroneous measurements. Several of these criteria are detailed in [33] and include, for example, a method for detecting poor fixturing of the instrument to the pipe by scanning load-depth curves for indications of excessive compliance.

### 2.2. Tensile Testing

Uniaxial tension tests were also conducted on many of the same pipe features on which IIT was conducted to provide benchmark YS and UTS values. In most cases, tensile tests were performed on both longitudinal and transverse samples. The tensile samples had nominal dimensions that conformed to Figure 3 in ASTM A370, including a gage length of 5.1 cm (2.0 in.) and a width of 3.8 cm (1.5 in.), as well as a sample thickness consistent with the pipe wall thickness, 4.8 mm (0.19 in.) to 19 mm (0.75 in.). Specimens also had some initial curvature since they were extracted from pipelines. The transverse specimens were flattened in a press prior to tensile testing. The longitudinal tensile specimens were not flattened prior to testing. An averaging extensometer, with a 5.1-cm (2-inch) gage length and 2.5-cm (1.0-inch) maximum elongation, was used to calculate average strain and to facilitate analysis of the full stress-strain response. Specimens were loaded in displacement control at a displacement rate of $3\times 10^{-3}$ cm/s (0.05 in/min), corresponding to a nominal strain rate of $3.7\times 10^{-4}\;s^{-1}$. After reaching 1% nominal strain, the displacement rate was increased by a factor of 10. Thus, the strain rate in uniaxial tension compared well with the estimated rate through IIT testing.

Throughout this work, the YS reported from tensile tests is the stress at 0.5% nominal strain. This definition is consistent with the definition adopted for IIT, but no power-law model is fitted to the tensile response; YS is read directly from the measured stress-strain response. The UTS from tensile tests is the true stress, corresponding to the load maximum.

### 2.3. Chemical Composition Testing

For the chemical composition results presented in this paper, combustion testing was used for carbon and sulfur, and OES was used for all other elements. Samples (approximately 2.5 cm × 2.5 cm) were cut out of the pipes and tested. For the combustion method, a drill was used to remove chips from the sample, and 2–4 combustion replicate measurements were taken. The OES data represent the average of 2–4 OES burns on the surface of the sample. In this work, only the elements manganese and carbon were used for strength prediction because these two elements are specified in [2] for all pipe grade specifications and are present in amounts large enough for reliable measurement through the NDT techniques currently under development [17,18]. Other elements, such as silicon, sulfur and the microalloying elements, vanadium, titanium, niobium, chromium, and molybdenum, require further validation of the NDT techniques but could be included in future work.

### 2.4. Materials

The samples in this study consisted of 197 steel pipe features, including line pipe and fittings. The feature materials included mostly traditional, low-carbon, moderate-strength steel, as well as 3–5 thermomechanically controlled processed (TMCP) steel pipes and 3–5 quenched and tempered (Q&T) steel pipes. As will be described below, the carbon content ranged from about 0.05% to 0.25% in the steels in this study. YS typically ranged between 275 and 415 MPa (40 and 60 ksi), and UTS was between 415 and 550 MPa (60 and 80 ksi). IIT measurements were obtained on site on either active or decommissioned pipeline segments, ranging in diameter from 10.2 cm (4 in.) to 91.4 cm (36 in.), and wall thickness from 4.8 mm (0.188 in.) to 19 mm (0.75 in.).

Prior to indentation, the pipeline surface was carefully prepared to a “mirror finish.” The surface was ground with successively finer-grit sandpaper, using a handheld mechanical sander to a final pass of 2000 grit. The orientation of the sander, relative to the surface, was changed with each successive pass to mitigate groove-in. This entire process is designed to remove a minimum of 10 μm of wall thickness from the OD and, therefore, minimize the presence of any decarburization layer that may exist in the pipe sample.

## 3. Results

### 3.1. IIT Measurements and Analysis

**Figure 4:**
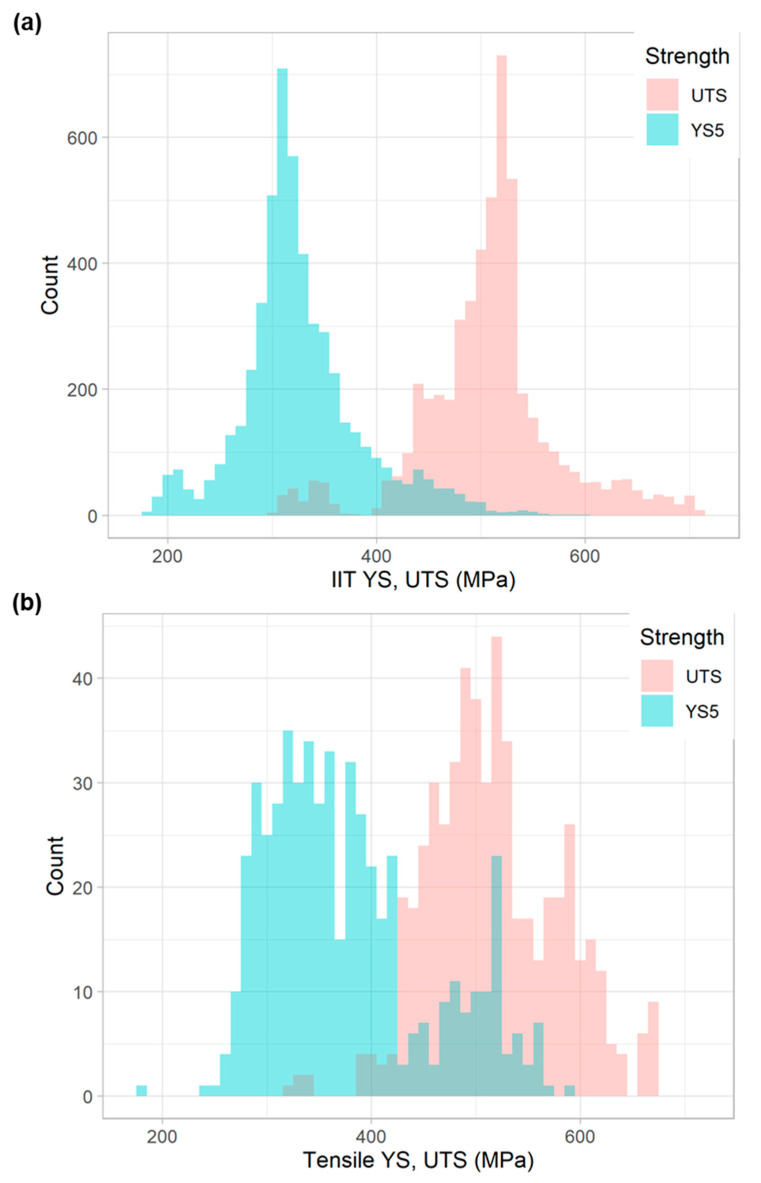
Histogram of YS and UTS for all (**a**) IIT and (**b**) tensile measurements in this study.

In total, 5231 IIT measurements were taken across 197 distinct pipe features. The full spectrum of YS and UTS values obtained from these measurements is shown in Figure 4a. YS values typically fall between 275 and 415 MPa (40 and 60 ksi), while most UTS values fall between 415 and 550 MPa (60 and 80 ksi), though the ranges do overlap to some degree.

**Figure 5:**
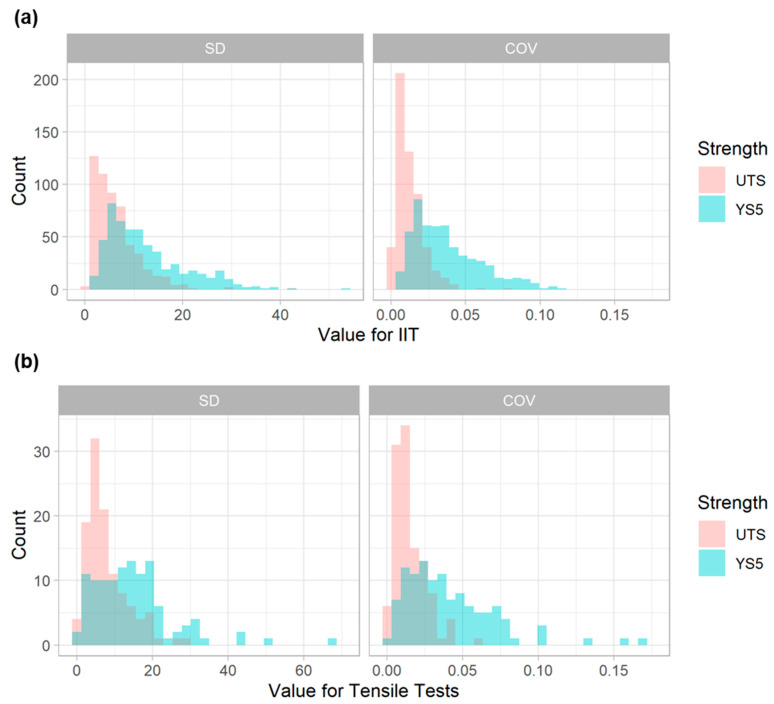
Histograms of SD (left) and COV (right) for (**a**) IIT and (**b**) tensile tests.

To quantify the variability in these measurements, IIT measurements were grouped according to the specific pipe feature and feature location from which each measurement was obtained. The mean, standard deviation (SD), and coefficient of variation (COV) were computed within each of these groupings. The COV, defined as the SD divided by the mean, provides a normalized measure of the variability. Histograms of SD and COV for YS and UTS, from IIT measurements, are presented in Figure 5a. These figures show that UTS tends to exhibit less variability than YS, both in absolute terms (indicated by SD) and relative terms (indicated by COV). The average SD for YS (12 MPa or 1.8 ksi) is twice that of UTS (6 MPa or 0.9 ksi), while the average COV for YS (0.037) is three-times greater than that of UTS (0.012).

**Figure 6:**
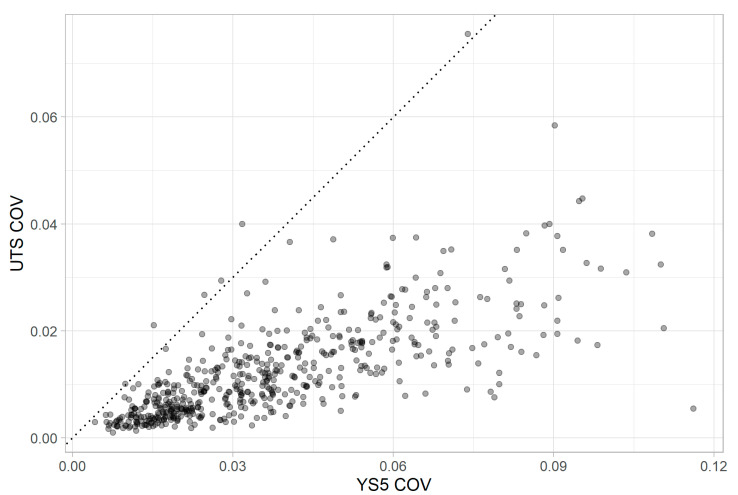
UTS COV vs. YS COV for IIT data.

To show how the variability of YS and UTS, from IIT measurements, directly compare within each grouping, the quantities’ COVs are plotted against each other in Figure 6. A unity line was also added to the figure for context. Points falling below the unity line indicate groupings where the COV for YS is greater than the COV for UTS, while points falling above the unity line indicate grouping where the COV for UTS is greater than the COV for YS. The trend shown by the histograms in Figure 5a is emphasized in Figure 6. The superior precision of the UTS measurements is clear: out of 544 test locations, only 6 of them had a COV for UTS that exceed the COV for YS.

### 3.2. Comparison to Destructive Data

With respect to tensile testing data, 531 tensile tests were executed across 136 distinct pipe features. The histogram of YS and UTS measurements, obtained from the tensile testing program, is shown in Figure 4b. YS values typically fell between 275 and 415 MPa (40 and 60 ksi) (though some values also fell between 480 and 550 MPa), while most UTS values fell between 415 and 620 MPa (60 and 90 ksi). Note that these tensile results in Figure 4b are plotted on the same x-axis scale as the IIT results in Figure 4a. Though the IIT results exhibit a better-defined peak than the tensile results, it is important to note that there were ten times more IIT measurements obtained than tensile measurements.

Histograms of SD and COV for YS and UTS, from the tensile measurements, are presented in Figure 5b. These values were computed for the tensile data in a manner identical to that of the IIT values: the measurements were first grouped by pipe section from which each measurement was obtained. The mean and SD were computed within each of these groupings, and the COV was also computed. As with the variability displayed by the IIT measurements, these figures show that UTS tended to exhibit less variability than YS, both in terms of SD and COV. Again, the tensile figures in Figure 5b are plotted with the same x-axis range as the IIT data in Figure 5a. This comparison suggests that the tensile measurements exhibited less variability than IIT.

**Figure 7:**
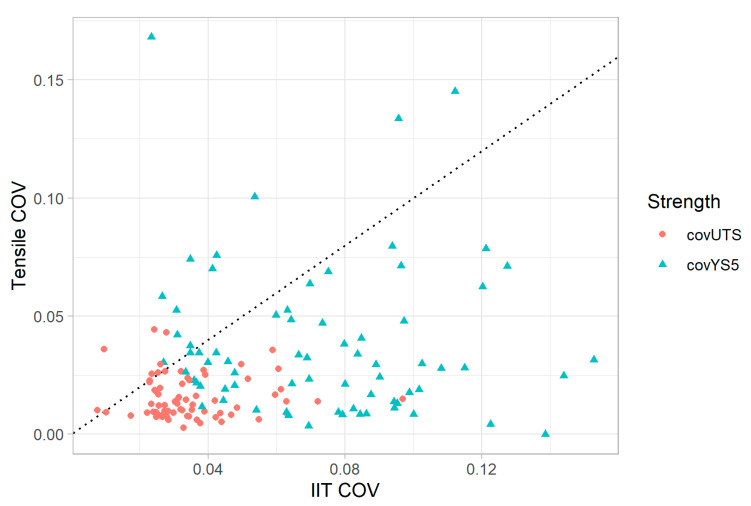
Tensile vs. IIT COV for both YS and UTS.

To compare the variability of IIT measurements with tensile testing measurements directly, a subset of the pipe features was analyzed where both IIT and tensile testing were performed. In total, 83 distinct pipe features underwent both tensile testing and IIT. Figure 7 presents a scatter plot comparing the relative variability (COV) of IIT to that of tensile testing for the pipe features where both were performed. Both YS (plotted as ▲) and UTS (plotted as ●) are shown. A unity line is also shown for context: points lying below the unity line indicate instances where the strength quantity measured by IIT had a higher COV than that from tensile testing, and points lying above the unity line indicate where the strength quantity, measured by tensile testing, had a higher COV. The data in Figure 7 indicates that IIT measurements consistently displayed greater relative variability than tensile testing, and COVs for YS were generally greater than COVs for UTS. Note that, because the calculation of COV requires more than one measurement, Figure 7 consists of 72 pipe features instead of 83 since only pipe features with more than one tensile test were considered.

**Figure 8:**
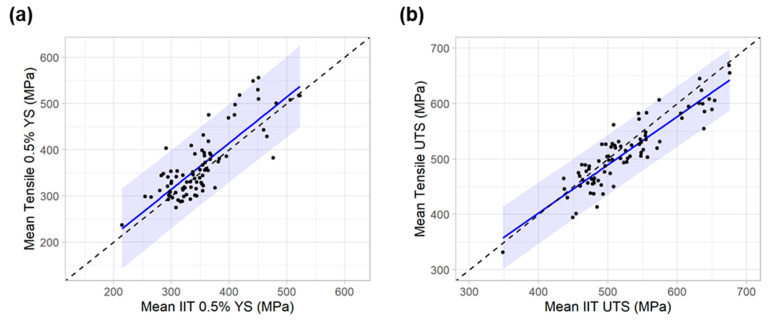
Unity plots comparing mean IIT and tensile measurements on a per-feature basis with linear regression (blue line) and 95% prediction interval (blue shaded region) for (**a**) YS and (**b**) UTS.

The next important comparison is shown in Figure 8, where the mean IIT YS and UTS for each pipe feature is plotted against the mean tensile YS and UTS. Figure 8a plots the mean IIT YS versus mean tensile YS (mean values are for the pipe feature tested). Figure 8b plots mean UTS values of IIT against tensile. A linear regression is also shown on both plots, along with the 95% prediction interval (shaded region). The regression for UTS exhibited a superior coefficient of correlation (R^2^) value than YS (0.81 vs. 0.67), as well as a lower root-mean square error (27 vs. 43 MPa or 3.9 vs. 6.2 ksi). The 95% prediction interval is also notably tighter for UTS, with a typical height of about 100 MPa (15 ksi) compared to 175 MPa (25 ksi) for YS.

These results collectively indicate that, overall, IIT measurements of UTS compare better to direct measurement through tensile testing than YS. Likewise, the relative robustness of UTS measurements compared to YS is not a phenomenon observed strictly in IIT measurements—the same finding exists within the tensile data as well. The results show that IIT consistently exhibits greater scatter than tensile testing and that UTS, for both test methods, exhibits more consistency and less scatter than YS.

### 3.3. IIT + Composition Machine Learning Model

Since the goal of this effort is to determine the mechanical properties of pipeline features without TVC records, it is naturally desirable to achieve the most accurate estimate of YS and UTS possible. While chemical composition is conventionally linked with microstructure and processing parameters to determine mechanical strength *a priori*, such an approach would be impossible for pipeline features lacking TVC records, since their manufacturing information is unknown. It was hypothesized, therefore, that chemical composition data could be combined with (nondestructive) IIT strength measurements to improve the correlation of the latter with (destructive) tensile strength.

Among all elements detected through OES and combustion, only carbon and manganese were selected for incorporation into a machine learning model. These two elements are specified in [2] for all pipe grade specifications, and they are present in amounts large-enough for reliable measurement through the NDT techniques currently under development. Figure 8 shows the feature-mean difference between IIT and tensile YS (the residuals) plotted against the measured carbon (C) and manganese (Mn) content in each feature. There are noticeable trends in the residuals for both elements that indicate that this composition data could be incorporated into a strength prediction model. Note that manganese and carbon have opposite signed slopes relative to increasing weight percent of the elements.

**Figure 9:**
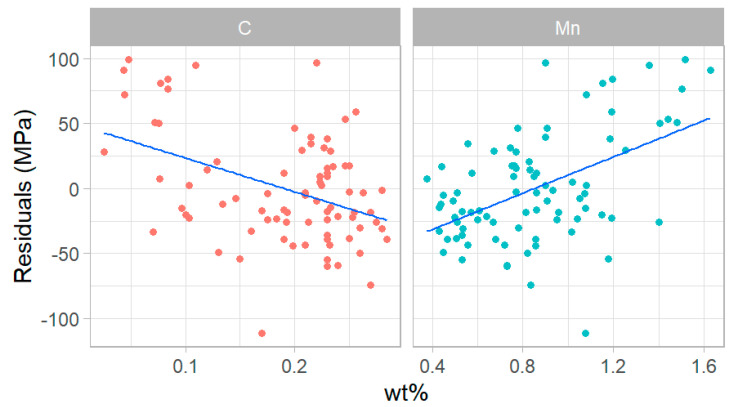
Error between mean IIT YS and mean tensile YS for each feature plotted against mass fraction manganese (wt.%) and mass fraction carbon (wt.%).

A least-squares regression model was selected for this analysis. Regression is a robust technique and is the most interpretable of all machine learning methods. It is also readily deployable: once the regression is complete, the numerical coefficients can be applied to new data without having to store a trained model. The analysis was completed with the open-source statistical programing language R, using the Tidymodels package [34]. The model definition included an interaction term between manganese and carbon (Mn × C) to account for their opposite-signed relationship, with residuals, seen in Figure 9. If this was not accounted for in the model, it would violate the assumption of independence of predictors since the effect of one would be dependent on the value of the other.

**Figure 10:**
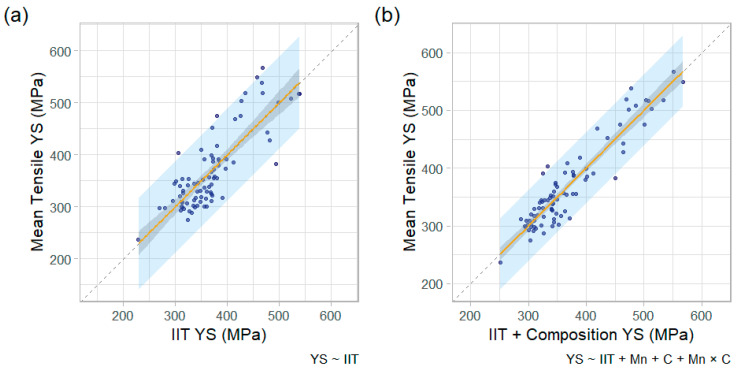
Unity plots comparing estimated versus observed YS with 95% confidence interval (gray shaded region) and 95% prediction interval (blue shaded region) for (**a**) IIT-only and (**b**) IIT + Composition.

Plots of the IIT-only YS and IIT + Composition YS, against the tensile YS, are shown in Figure 10 (note that the data presented in Figure 10a is the same as that shown in Figure 8a). By graphical inspection, the model that includes chemical composition (C and Mn content) appears more accurate than IIT-only. The improvement is quantified by the parameters shown in Table 2. The linear regression’s adjusted R^2^ improved from 0.67 to 0.87 with the inclusion of carbon and manganese content, and the root-mean square error (RMSE) improved from 43 MPa (6.2 ksi) to 27 MPa (3.9 ksi). The adjusted R^2^ is a modified version of the regular R^2^ correlation coefficient that increases when the additional predictors improve the model by more than chance. Thus, it is a better metric for comparing models of varying complexity to determine if additional predictors are improving the model performance rather than over-fitting the model to statistical noise.

Additional numerical parameters pertaining to the IIT + Composition YS model are given in Table 3. To make the intercept coefficient more meaningful and remove the effects of correlated variables, the predictors were centered (subtracting the mean of each). Thus, the resulting regression equation includes the subtraction of the mean for each predictor so that it can be applied with an arbitrary data set. The resulting regression equation is:

(10) $$\mathrm{YS}\left(\mathrm{MPa}\right)=364+0.455IIT_{\mathrm{YS},ctr}+105{\mathrm{Mn}}_{ctr}-215C_{ctr}-451{\mathrm{Mn}}_{ctr}\times C_{ctr}$$

wherein ${\mathrm{Mn}}_{ctr}=\mathrm{Mn}-0.851$, $C_{ctr}=C-0.190$, $IIT_{\mathrm{YS},ctr}=IIT_{\mathrm{YS}}-352$, and $IIT_{\mathrm{YS}}$ is the yield strength estimate by IIT (MPa). Of the computed regression coefficients for each parameter, none are zero or near-zero. A zero or near-zero coefficient would indicate that a predictor had no effect on the model. The *p*-value is also shown, which is the probability of observing the results, under the null hypothesis, that the coefficient is equal to zero for the input variable. A *p*-value of less than 0.05 indicates the probability that the observed changes in the predicted variable, due to chance, are 5% or less with the inclusion of the predictor variable. Thus, with all *p*-values well below 0.05, we confidently judge that the new chemical composition predictors were significant. The significance of the input variables is also indicated by the fact that the range of confidence intervals exclude zero for all variables.

**Figure 11:**
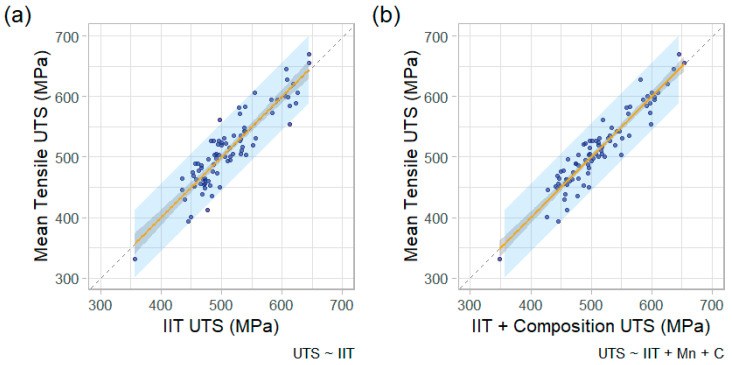
Unity plots comparing estimated versus observed UTS, with 95% confidence interval (gray shaded region) and 95% prediction interval (blue shaded region) for (**a**) IIT alone and (**b**) IIT + composition.

A similar regression model was developed for UTS and compared to an IIT-only model:

(11) $$\mathrm{UTS}\left(\mathrm{MPa}\right)=512+0.611IIT_{\mathrm{UTS},ctr}+87.6{\mathrm{Mn}}_{ctr}+119C_{ctr}$$

wherein ${\mathrm{Mn}}_{ctr}=\mathrm{Mn}-0.851$, $C_{ctr}=C-0.190$, $IIT_{\mathrm{UTS},ctr}=IIT_{\mathrm{UTS}}-525$, and $IIT_{\mathrm{UTS}}$ is the ultimate tensile strength estimate by IIT (MPa). Table 4 shows the respective models’ coefficients. The estimated UTS values for the two models are plotted against the tensile (destructive) UTS values, side-by-side, in Figure 11. Note that the “IIT Only” model presented in Figure 11a is identical to the unity plot presented in Figure 8b. The inclusion of manganese and carbon content in the model improved the adjusted R^2^ for UTS estimates from 0.81 to 0.88, which is an 8% improvement. This 8% improvement for UTS compares to a 29% improvement in adjusted R^2^ in the YS models. Incorporation of chemical composition improved the RMSE of UTS by 20%, which contrasts to a 36% improvement in the YS that was achieved.

Additional numerical parameters pertaining to the IIT + Composition model for UTS are given in Table 5. As in Table 3, the *p*-values are all well below 0.05, indicating that each predictor makes a significant contribution to the model. A noticeable difference from the YS model is that the carbon coefficient is positive here, indicating that an increase in carbon correlates with increased UTS. However, in the YS model, carbon had a negative coefficient. Carbon also has a relatively wide confidence interval with a lower limit near zero. Finally, it is worth noting that, since carbon and manganese are positively correlated here, the use of an interaction term was not necessary in the UTS model.

In general, the inclusion of carbon and manganese content into a regression model, along with IIT strength measurements, improved the accuracy of the strength estimates relative to the tensile measurements. YS values benefitted from chemical composition much more than UTS. This result demonstrates the robustness of IIT UTS measurements compared to YS measurements. However, since each element chosen for this modeling plays a different role in yield and tensile strength, the exact mechanism behind the observation remains unclear.

### 3.4. An Example Measurement

To illustrate how these models might be applied in practice, we briefly present data from a representative pipe feature that underwent tensile, IIT, and chemical composition testing. The selected sample was chosen because (1) its mechanical properties were close to the average mechanical properties of all materials examined in this study, and (2) a relatively large number of replicate measurements were taken.

**Figure 12:**
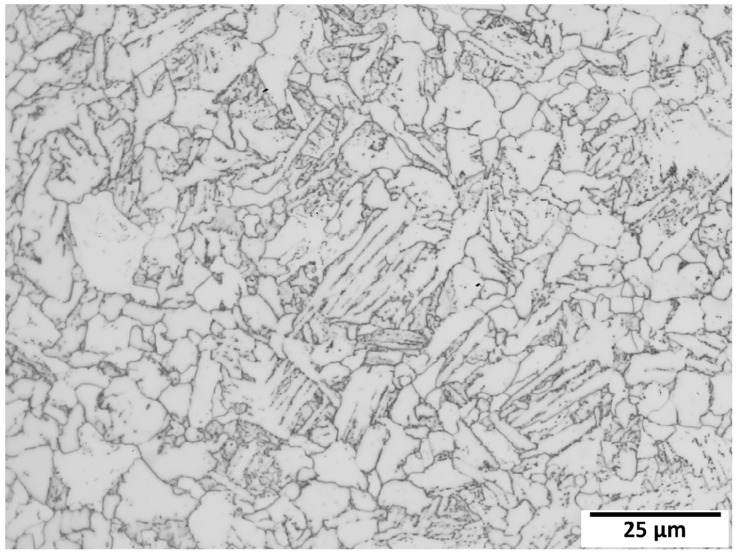
Micrograph of the cross section (longitudinal orientation; 1000×) of the 16-inch (41 cm) OD pipe; Q&T API-5L X42Q line pipe steel. For reference, the indenter diameter is 500 μm, while the image shown is approximately 135 μm wide.

The microstructure of this sample is shown in Figure 12, and it is typical of a quenched and tempered (Q&T) API-5L X42Q line pipe steel. This photomicrograph was produced by destructive sectioning, polishing, etching, and image capture of the sample’s axial-radial-oriented face at an original magnification of 1000×. The steel was austenitized at ~910 °C, then quenched, and tempered at ~660 °C. The microstructure is similar to the Q&T S690 steel, tempered at 660 °C, which is illustrated in [35].

**Figure 13:**
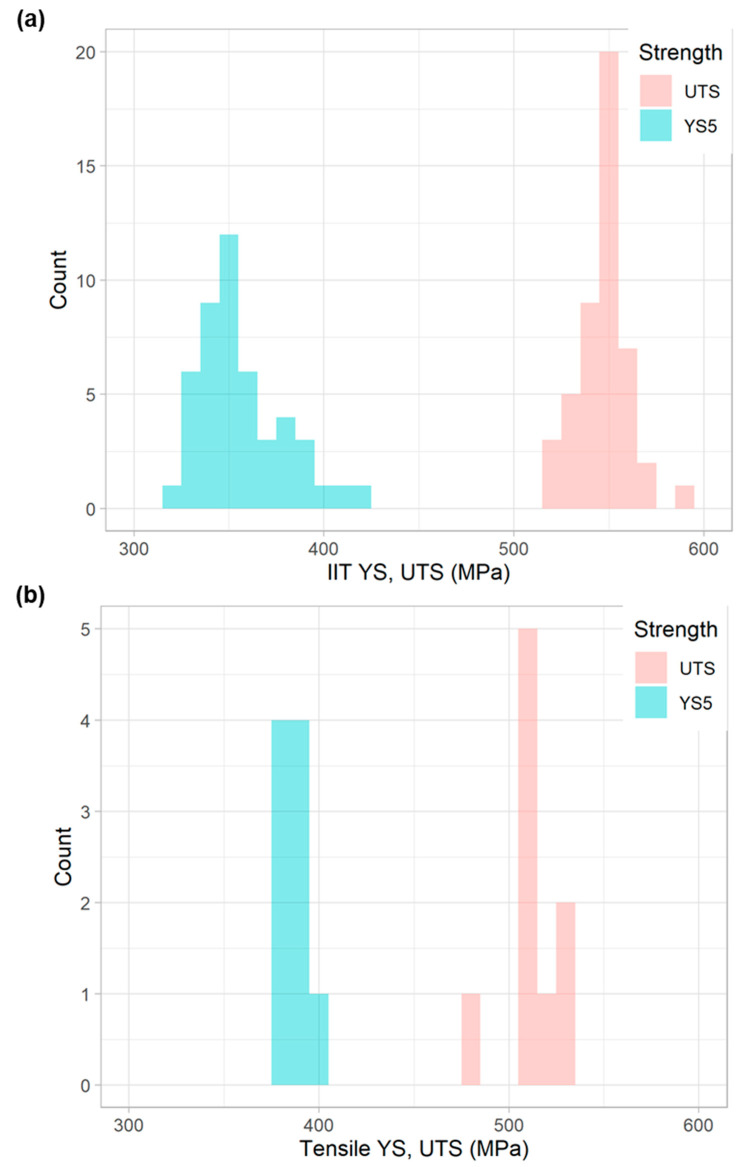
Histogram of YS and UTS for (**a**) IIT and (**b**) tensile measurements performed on the 16-inch (41-cm) OD pipe in the presented example.

Two longitudinal and seven transverse samples from this pipe underwent tensile tests, followed by 47 replicate IIT measurements. Since the pipe had an OD of 16 inches (41 cm), the roller chain mounting fixture was used. The distribution of tensile and IIT measurements of YS and UTS are shown in Figure 13. IIT YS values range from approximately 320 to 420 MPa (46 ksi to 62 ksi), while UTS values range from approximately 515 to 585 MPa (75 ksi to 85 ksi). Tensile YS values range from approximately 375 to 400 MPa (54 ksi to 57 ksi), while UTS values range from approximately 480 to 525 MPa (70 ksi to 76 ksi). Mean and SD values, for both IIT and tensile measurements, are shown in Table 6.

Based on the mean measurement value from IIT (357 MPa), the IIT-only model shown in Section 3.3 results in a YS estimate of 373 MPa (54 ksi) with a 95% prediction interval range of 288 to 459 MPa (42 ksi to 67 ksi). The model gives a UTS of 532 MPa (77 ksi) with a 95% prediction interval range of 479 to 586 MPa (69 ksi to 85 ksi). Table 6 summarizes these results.

Chemical composition tests yielded measurements of 0.12 wt.% carbon and 0.83 wt.% manganese in this material. Other elements included 0.29 wt.% silicon, 0.013 wt.% phosphorus, 0.004 wt.% sulfur, 0.11 wt.% chromium, 0.14 wt.% nickel, 0.24 wt.% copper, 0.02 wt.% molybdenum, and 0.03 wt.% vanadium, and the balance was iron (>98%). Using the IIT + Composition regression models established in Section 3.3 (Equations (10) and (11)), the estimated YS and UTS are 378 MPa (55 ksi) and 516 MPa (75 ksi), respectively. Likewise, the 95% prediction interval range is 323 MPa to 434 MPa (47 ksi to 63 ksi) for YS, and 473 MPa to 559 MPa (69 ksi to 81 ksi) for UTS (see Table 6).

Overall, by using the IIT + Composition model in Equation (10), the difference between the estimated YS and mean tensile YS for this pipe sample material was improved to 9 MPa from 14 MPa compared to the IIT-only model—a 36% decrease. In addition, the 95% prediction interval range for YS was reduced by approximately 35%. With respect to UTS, using the IIT + Composition model in Equation (11) results in a difference between the estimated UTS and mean tensile UTS of 5 MPa, which is a 76% reduction compared to the difference using the IIT-only model (21 MPa). Likewise, the 95% prediction interval range for UTS was reduced by approximately 20%.

## 4. Discussion

The data is quite clear: IIT provides robust measurements of UTS, especially compared to measurements of YS on the same pipe features. UTS exhibited less variability than YS in both a relative sense (COV) and absolute sense (SD). We attribute this finding to variety of factors: experimental, mechanical, and metallurgical.

### 4.1. Experimental Factors

Figure 7 showed that UTS was less variable than YS in replicate tensile tests as well as IIT. Thus, it appears that the relative performance found in the IIT data set is, in fact, not unique to IIT. However, there may be aspects of the IIT algorithm that further contribute to this result.

**Figure 14:**
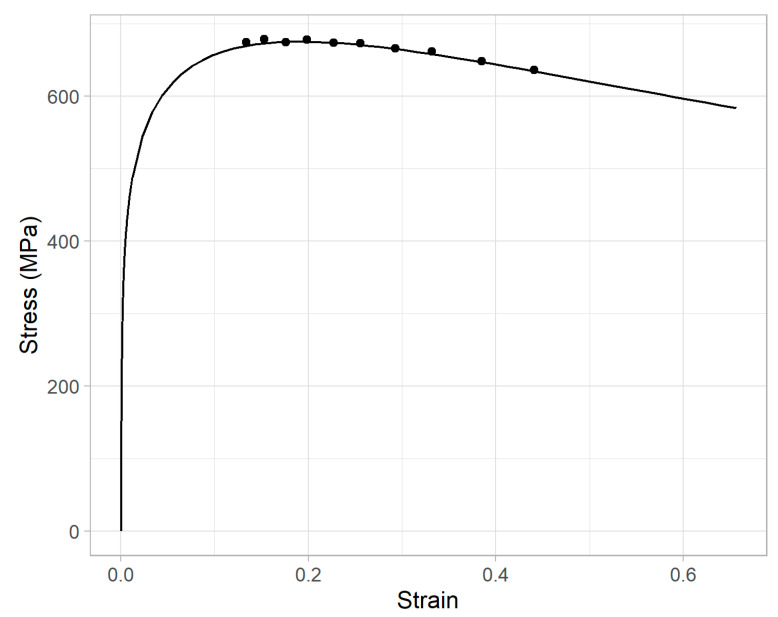
Nominal stress-strain, from the 6th through 15th IIT load maximum points, and computed power-law curve.

Recall from Section 2.1 and Figure 2 that a power-law hardening curve is fit to the stress and strain corresponding to the final 10 of the 15 load maximum points. Figure 14 shows the final computed power-law stress-strain curve from a representative IIT measurement, along with the discrete values of stress and strain from those final 10 load maximum points. Note that nominal stress and strain are plotted here for better visualization of the UTS. The figure shows that the UTS occurs within the discrete points. In contrast, the YS (the stress at a strain of 0.5%) is reached well before any of the discrete points. In fact, the lowest of the discrete stress-strain points, to which the power-law was fit, has a strain of about 0.12. Therefore, obtaining the YS from this power-law stress-strain curve requires a substantial extrapolation beyond the data to which the power-law was fit. In contrast, the UTS is a direct interpolation. Small variations in the computed power-law parameters K or n could, therefore, have a much stronger impact on YS than they would on UTS.

We further note that this extrapolation also contributes to the inferior R^2^ and RMSE of the IIT-vs-tensile comparison of YS measurements in Figure 8, compared to the UTS measurements unity plot. Many of the materials tested in this work exhibited yield-point elongation (YPE), or a stress plateau, following the elastic limit in tensile tests. A power-law curve, fit to the hardening portion of the stress-strain response and extrapolated to 0.5% strain (inside the stress plateau), would lead to an underestimation of the stress at that strain level. While YPE is not detectable in the IIT measurements we have obtained, its existence in some of the tensile test data confounds the direct comparisons.

Among the three contributing factors discussed in this section, this experimental factor is considered less fundamental to the observed differences in YS and UTS measurements. As shown in Figure 7, similar trends were observed in data from our tensile testing program. Thus, the propensity for YS to exhibit greater variability than UTS seems to transcend this extrapolation effect that is unique to IIT. In contrast, the mechanical and metallurgical factors described below contribute to both tensile and IIT results. It would appear, then, that they are more significant in their contributions.

### 4.2. Mechanical Factors

One factor that likely contributes to the observation of less scatter in UTS than YS, for both IIT and tensile measurements, is that the tangent modulus, or instantaneous strain hardening rate, at yield is far greater than it is at UTS. The flow stress in a material approaching UTS, therefore, is less sensitive to small variations in strain than it is around yield. This effect can be shown quantitatively.

The tangent modulus for a power-law stress-strain relationship is given by:

(12) $$\frac{d\sigma }{d\epsilon }=Kn\epsilon^{n-1}$$

Recall the Considère analysis of an incompressible, power-law material under uniaxial tension results in the following prediction:

(13) $$\epsilon_{\mathrm{UTS}}=n$$

Therefore, combining Equations (12) and (13), the tangent modulus at UTS is:

(14) $${\frac{d\sigma }{d\epsilon }|}_{\mathrm{UTS}}=Kn\;n^{n-1}$$

At a strain of 0.5%, where YS is defined in this study, the tangent modulus is:

(15) $${\frac{d\sigma }{d\epsilon }|}_{\mathrm{YS}}=Kn{\left(0.005\right)}^{n-1}=Kn\;{\left(200\right)}^{1-n}$$

The ratio of tangent moduli at yield and UTS is therefore given by:

(16) dσdϵ|YSdσdϵ|UTS=(200)1−nnn−1=(200 n)1−n

For values of strain hardening exponent *n* between 0.2 and 0.3, as is typical for steels assessed in this work, the ratio of the tangent modulus varies between 17.6 and 19.2. It can be shown that this ratio reaches a maximum value of 19.2 when *n* takes on a value of 0.211. This important result can be interpreted to indicate that the YS is 17 to 19 times more sensitive to small variations in strain than the UTS. This sensitivity difference likely drives some of the observed robustness of UTS compared to YS observed in the measurements.

### 4.3. Metallurgical Factors

Regarding the greater uncertainty of YS versus UTS, many low-to-mid carbon steel samples exhibit an abrupt drop at the end of the elastic region of the stress-strain response called yield point elongation (YPE). The British Steel Corporation showed that YPE is caused by strain aging, which is the time-and-temperature-dependent migration of nitrogen and carbon atoms to dislocations [19]. These atoms have a “pinning” effect on the dislocations, thus raising the critical resolved shear stress for dislocation motion. The YPE, then, is the aggregate effect of sequentially “unpinning” these locally-pinned dislocations by raising the local critical resolved shear stress until a sufficient density of dislocations are available for bulk uniform plastic deformation. The consequence is a stress plateau observed around yielding in most steels, and a noisy stress-strain response for an unpredictable duration of plastic deformation (ranging from a fraction of a percent strain to several percent strain). After YPE, typical power-law work hardening usually ensues.

The line pipe steels in this study varied in terms of the existence and magnitude of YPE effects, which caused noise in the tensile YS data and degraded the ability to correlate tensile YS to IIT YS. It remains unclear how YPE manifests in the triaxial, compressive stress state beneath the indenter in IIT, but it is possible that YPE results in similar experimental difficulties in the IIT results as well. Certainly, the adoption of a power-law stress-strain model for IIT would not properly capture this behavior, thus leading to greater differences between IIT and tensile measurements of YS. The use of a 0.5% elongation-under-load definition for YS further contributes to the error associated with YPE. If the causes of YPE can be better understood, including the effects of YPE on IIT, then it could lead to a more accurate measurement of YS from IIT.

The equation for YS from the linear models, Equation (10), indicates that tensile YS is not solely dependent on IIT YS. Specifically, the coefficient in the equation for $IIT_{\mathrm{YS},ctr}$ is 0.455 (whereas the coefficient would be 1.0 for a perfectly one-to-one relationship between IIT YS and tensile YS). Compensating aspects include an intercept of 364 MPa, a positive coefficient associated with manganese content, and negative coefficients associated with carbon and manganese multiplied by carbon content. These indicate that the higher the manganese content in the steel, the more likely IIT is to underestimate the YS. Conversely, reduction in carbon in the steel would also result in underestimation of YS by IIT. The existence of these manganese and carbon terms may partially be due to the lack of a term for grain size, which was important in Equation (1). Aside from the inverse correlation of carbon content with strength via grain size, high YS steels tend to have lower carbon contents because of the negative effects of carbon on weldability and toughness [36]. Thus, the role of carbon could be indirectly predictive of YS.

Similarly, the equation for UTS from the linear models, Equation (11), indicates that the tensile UTS is not solely dependent on IIT UTS. The coefficient in the equation for $IIT_{\mathrm{UTS},ctr}$ is 0.611 (rather than 1.0 for a 1:1 relationship). There is an intercept of 512 MPa, and positive coefficients associated with manganese content. Again, these indicate that the higher the manganese content in the steel, the more likely IIT is to underestimate the UTS. Interestingly, however, whereas the carbon coefficient is negative in Equation (10), the carbon coefficient in Equation (11) is positive. This result indicates that the higher the carbon content, the more likely IIT is to underestimate the UTS. There is uncertainty, however, in the role of carbon as highlighted by the large range of the confidence interval for the carbon coefficient for UTS in Table 5 (38.3 to 200 per wt.% C). Apparently, the removal of carbon from the IIT + Composition model for UTS would cause little degradation of the model performance. Therefore, it is likely that the machine learning model derives more utility from the manganese content since it relies very little on the carbon content. Nevertheless, in this sizable dataset, a slight improvement exists with the inclusion of carbon, which is useful for improving strength estimates.

Incorporation of chemical composition into the machine learning model had a greater impact on improving the YS estimates than it did on improving the UTS estimates. Thus, we must consider how composition (specifically manganese and carbon content) could affect YS differently from UTS. Note that the machine learning model is indifferent to the physical mechanisms but could reveal interesting and useful correlations that could be related to causative physical mechanisms. For example, the utility of carbon in the regression model could stem from a correlative mechanism (as opposed to causative mechanism), such as that between carbon content and grain size. Recalling Equations (1) and (2), grain size has been shown to correlate with YS and UTS itself. Thus, although the carbon content changes do not control YS, carbon content could correlate with some other variable, such as grain size, that does affect YS. It is also possible that the regression model is utilizing the elemental variables to compensate for fundamental differences between monotonic tensile tests and indentation tests. The load application and stress state in the deformed material are fundamentally different, and the effects of these differences are unclear. However, the model could be taking advantage of the unique effects of the compositional elements on the work hardening behavior to improve the YS and UTS predictions.

## 5. Conclusions

IIT is an established technique for nondestructively estimating the mechanical properties of materials. PG&E, in response to recent federal regulatory changes, has undertaken a large pipeline materials verification effort, and IIT is a key tool in this work. In the past five years, PG&E has performed over 5000 IIT measurements across nearly two-hundred unique pipeline features. These measurements were performed with the Frontics AIS 2100 IIT instrument using a 0.5-mm diameter spherical indenter.

Results from this effort indicate that IIT’s estimates of UTS exhibit less variability than YS. Both the SD and COV, associated with replicate measurements on the same pipe location, are lower for UTS than YS. Thus, UTS exhibits less variability in both an absolute and relative sense. Interestingly, a similar trend was observed in tensile testing performed on some of the same pipe samples. This likely stems from the difference in tangent moduli between YS and UTS, hence the sensitivity of the stress to small changes in strain. Towards verifying the IIT measurements from this work, YS and UTS measurements, via IIT and tensile testing, were directly compared. The linear regression between IIT and tensile measurements for UTS exhibited a superior coefficient of correlation (R^2^) value than YS (0.81 vs. 0.67), as well as a lower root-mean square error (27 vs. 43 MPa, or 3.9 vs. 6.2 ksi). Thus, IIT’s measurements of UTS are not only less variable, but also more-accurately reflect tensile values, than YS.

IIT measurements were next combined with chemical composition data, which was collected as part of this project’s materials verification work. Specifically, multivariate linear regressions combining IIT (YS and UTS separately), carbon, and manganese were performed. Both YS and UTS estimates improved relative to the tensile measurements by combining IIT with these additional data. The YS estimate improved much more so than the UTS estimate, but the exact mechanisms behind this result remain unclear. Additional work is needed to clarify whether the observed improvements were due to causal or correlative factors.

As IIT data collection or post-processing practices advance, the observed difference in YS and UTS may change. For example, implementation of an automated outlier-detection algorithm that discards erroneous measurements may alter this trend in YS and UTS. The adopted IIT algorithm itself could, perhaps, be reevaluated as well. Extrapolation of YS in the IIT algorithm is necessary in the current technique due the mechanical sensitivity of the load cell and materials at low load during the first five load cycles. Evaluating alternative algorithms that do not require such large extrapolation would help in fully evaluating this behavior and, therefore, remains a longer-term objective.

With respect to using IIT and chemical composition data to determine strength, additional studies are needed in this area to fully understand the role of chemistry and strength and how they might behave in models that combine IIT data. For example, nitrogen content was not measured in these materials, and it may be possible to correlate between elements such as manganese and the reduction in nitrogen content. Thus, model accuracy could perhaps be improved by using the content of elements that may not be phenomenologically responsible for the changes in mechanical properties, but are instead correlated, with the responsible elements.

## Figures and Tables

**Table 1:** Numerical constants used in Equation (7) (values from [29]).

ω	*a*	*b*_1_	*b*_2_	*c*_1_	*c*_2_
0.75	0.131	−3.423	0.079	6.258	−8.072

**Table 2:** Comparison of metrics for the YS models.

Model	Adjusted R^2^	RMSE (MPa)
IIT Only	0.67	42.7
IIT + Composition	0.87	27.2

**Table 3:** YS IIT + Composition model; regression coefficients and parameters (coefficients appear in Equation (10)).

	Estimate	*p*-Value	Conf. Int. (Lower Lim.)	Conf. Int. (Upper Lim.)
(Intercept)	364	<1 × 10^−16^	357	370
YS (IIT)	0.455	8.45 × 10^−9^	0.315	0.595
Manganese	105	3.73 × 10^−11^	77.6	132
Carbon	−215	1.76 × 10^−4^	−324	−106
Mn × C	−451	1.84 × 10^−3^	−730	−173

**Table 4:** Comparison of metrics for the UTS models.

Model	Adj. R^2^	RMSE (MPa)
IIT Only	0.81	26.9
IIT + Composition	0.88	21.4

**Table 5:** UTS IIT + Composition model; regression coefficients and model parameters (coefficients appear in Equation (11)).

	Estimate	*p*-Value	Conf. Int. (Lower Lim.)	Conf. Int. (Upper Lim.)
(Intercept)	512	<1 × 10^−16^	507	516
UTS (IIT)	0.611	1.76 × 10^−15^	0.488	0.734
Manganese	87.6	2.64 × 10^−9^	61.6	114
Carbon	119	4.39 × 10^−3^	38.3	200

**Table 6:** YS and UTS estimates based on the regression models presented in Section 3.3, including corresponding mean tensile and IIT measurements, for the 16-inch (41-cm) OD pipe in the presented example.

	YS (MPa)	YS Uncertainty * (MPa)	UTS (MPa)	UTS Uncertainty * (MPa)
Mean Tensile Measurement	387	8.3	511	13.6
Mean IIT Measurement	357	22.6	547	13.5
IIT-Only Trained Estimate	373	288–459	532	479–586
IIT+Composition Trained Estimate	378	323–434	516	473–559

* Note: Uncertainty values for measurements are represented as the standard deviation, while uncertainty values for estimates are represented as the range of the 95% prediction interval.

## Data Availability

The data presented in this study are available on request from the corresponding author. The data are not publicly available due to concerns related to security of critical infrastructure.
